# Cost-effectiveness of Microsoft Academic Graph with machine learning for automated study identification in a living map of coronavirus disease 2019 (COVID-19) research

**DOI:** 10.12688/wellcomeopenres.17141.2

**Published:** 2024-03-26

**Authors:** Ian Shemilt, Anneliese Arno, James Thomas, Theo Lorenc, Claire Khouja, Gary Raine, Katy Sutcliffe, D'Souza Preethy, Irene Kwan, Kath Wright, Amanda Sowden

**Affiliations:** 1 EPPI-Centre, UCL Social Research Institute, University College London, London, London, WC1H 0NR, UK; 2 Centre for Reviews and Dissemination, University of York, UK, York, Yorkshire, UK

**Keywords:** Automation, systematic review, machine learning, evidence synthesis, systematic map

## Abstract

**Background:**

Identifying new, eligible studies for integration into living systematic reviews and maps usually relies on conventional Boolean updating searches of multiple databases and manual processing of the updated results. Automated searches of one, comprehensive, continuously updated source, with adjunctive machine learning, could enable more efficient searching, selection and prioritisation workflows for updating (living) reviews and maps, though research is needed to establish this. Microsoft Academic Graph (MAG) is a potentially comprehensive single source which also contains metadata that can be used in machine learning to help efficiently identify eligible studies. This study sought to establish whether: (a) MAG was a sufficiently sensitive single source to maintain our living map of COVID-19 research; and (b) eligible records could be identified with an acceptably high level of specificity.

**Methods:**

We conducted an eight-arm cost-effectiveness analysis to assess the costs, recall and precision of semi-automated workflows, incorporating MAG with adjunctive machine learning, for continually updating our living map. Resource use data (time use) were collected from information specialists and other researchers involved in map production. Our systematic review software, EPPI-Reviewer, was adapted to incorporate MAG and associated machine learning workflows, and also used to collect data on recall, precision, and manual screening workload.

**Results:**

The semi-automated MAG-enabled workflow dominated conventional workflows in both the base case and sensitivity analyses. At one month our MAG-enabled workflow with machine learning, active learning and fixed screening targets identified 469 additional, eligible articles for inclusion in our living map, and cost £3,179 GBP per week less, compared with conventional methods relying on Boolean searches of Medline and Embase.

**Conclusions:**

We were able to increase recall and coverage of a large living map, whilst reducing its production costs. This finding is likely to be transferrable to OpenAlex, MAG’s successor database platform.

## Introduction

When the evidence base is evolving quickly, as in the case of COVID-19, it is important that decision-makers can draw on the latest research through continuously updated (living) maps and systematic reviews. Unfortunately, due to the sheer number of papers being published, and the lack of specificity available in online databases for identifying relevant research, maintaining living maps and reviews up to date is a resource-intensive process. Improving the efficiency of evidence synthesis production workflows is thus an important catalyst to enabling better health decisions and outcomes. Finding sufficiently reliable, but also less costly, ways to identify and classify studies at scale has become an active area of methods research and methodological development, with the potential to help reduce waste in research and reduce research costs worldwide.

Study identification comprises searching and selection (screening) tasks. While an array of ‘human-in-the-loop’ automation tools has been developed to support these tasks ^1^, uptake has so far been limited ^2^. Previous research has highlighted that automation tools need to have a relative advantage when compared with conventional tools and methods if they are to become widely diffused and adopted into practice ^3, 4^. For screening, a critical issue is demonstrating that semi-automated workflows will maintain or improve upon the recall (sensitivity) of current practice. Researchers undertaking evidence synthesis are justifiably averse to ‘missing’ eligible studies (i.e. decreased recall), especially when this might reduce the credibility, reliability and/or certainty of the published findings.

### Automating search tasks

To date, searching for eligible articles has relied primarily on information specialists conducting updated Boolean searches across multiple databases, and then manually processing the retrieved bibliographic records, including deduplication across those multiple sources. This is partly because bibliographic records have, up until recently, largely been reposited in closed-access, proprietary databases with some duplication between them, but with each also possibly containing records not found elsewhere. However, the idea that bibliographic records should be treated as ‘commercial property’ in this way is starting to erode, since it runs contrary to the principles of open science and undermines the value and impact of (publicly and privately funded) research. This erosion can be seen in recent, currently ongoing efforts to index all the world’s research in Microsoft Academic and Google Scholar databases, alongside the increasing availability of records that were once ‘closed’ in the open-access CrossRef and ‘OpenCitation’ repositories. (See, for example, the relatively recent announcement that Elsevier is opening its bibliographic citation dataset.) Increasing openness of bibliographic data, combined with ‘web scale’ datasets of vast numbers of records, opens new, transformational opportunities for locating research at scale. If most research is available in a single, comprehensive dataset, then the focus of information specialists’ work could shift towards developing and curating precise searches of that dataset, possibly using machine learning tools that are not available in commercial databases, with the potential for large overall efficiency gains.

Microsoft Academic Graph (MAG) is a large open-access dataset and repository that comprises over 250 million bibliographic records of scientific research articles ^5^. Microsoft makes the entire MAG dataset available for third-party use under a creative commons license. During the early part of the COVID-19 pandemic, until November 2020, an updated version of the MAG dataset was released every 7 to 10 days; since then, it is updated every 14 days. A key feature of the MAG dataset is that its bibliographic records are all connected in a large network graph of conceptual, citation and other relationships. This provides an opportunity to develop tools that exploit network graph features, alongside text features, of records, to help expedite the searching process. Microsoft has recently announced the closure of Microsoft Academic. Fortunately, the organisation *Our Research* has announced a successor called ‘OpenAlex’ and is liaising with Microsoft to ensure it has comparable coverage. The Allen Institute for AI is also planning to expand its open access dataset in Semantic Scholar, so while the closure of Microsoft Academic is a major loss to open science, the move towards increasing availability of open access bibliographic information seems set to continue. [Update November 2022: Microsoft Academic closed at the end of 2021 and the OpenAlex team has picked up its role and it is now the single largest open access repository of scientific bibliographic data, having incorporated the MAG dataset and started to maintain it as part of the successor OpenAlex dataset, which is organised using essentially the same network graph structure. The results reported here for MAG are therefore highly likely to be transferrable to OpenAlex. Our EPPI-Reviewer software (see below) switched over from MAG to use OpenAlex as its key source of data in January 2022.]

The MAG (and OpenAlex) datasets are published in the form of (very) large text files that can be imported into database software. They are not suitable for utilisation on desktop PCs by mainstream systematic reviewers though, due to their size and the fact that they do not come with software that provides a means to search them. They do, however, open new possibilities for developers for two reasons. First, search functionality need not be limited according to the tools that, for example, OVID uses to publish its databases. For example, features that are more attuned to the systematic review context can be developed using new technologies such as machine learning. Second, if these sources are sufficiently comprehensive, it may be possible to completely automate searching (and deduplication) for maintaining living systematic reviews and maps, as these use cases can make use of pre-existing data on which to train machine learning models.

Research is needed to investigate the extent to which using datasets such as MAG as a single source can improve the efficiency of study identification for other living maps, registers, systematic reviews and tertiary databases of research. We have therefore been actively developing a suite of tools to enable automated searching of a local copy of the MAG dataset in EPPI-Reviewer, web browser-based systematic review software that is hosted by UCL ^1^. These tools include a novel machine learning-based recommender model for continuous evidence surveillance (the 'AutoUpdate' model) ^6^, developed in collaboration with Microsoft™ to support the Human Behaviour-Change Project ^7^ and evidence surveillance activities across the EPPI-Centre. The AutoUpdate model is trained to infer the relevance of newly published MAG records to existing living maps, registers, systematic reviews and databases of research articles that subscribe to it. This is based on a supervised dataset, comprising graph and text features from MAG records previously selected for inclusion in those resources. A ‘custom Boolean search’ feature is also available, enabling searches of the updated MAG dataset using more conventional Boolean-type search strategies. This suite of tools is being used to support a portfolio of methods research projects investigating use of the MAG dataset as a single source for study identification in systematic reviews and related use scenarios. [Update November 2022: the above tools were migrated to utilise the OpenAlex dataset through 2022.]

### Automating study selection tasks when updating maps and reviews

Selecting eligible articles has conventionally relied on manual screening of all unique bibliographic records, retrieved from updated searches, against pre-specified criteria. Two potential approaches to automating the study selection task involve: (1) Identifying and discarding ineligible records prior to manual screening, while retaining eligible records; and (2) Prioritising (retained) eligible records for manual screening, while deprioritising (retained) ineligible records. These two approaches can be applied individually or in sequence. Machine learning (ML) tools that enable both approaches have been under active development in systematic review tools for some years and made available in select systematic review software, including EPPI Reviewer ^1, 8^. For (1), binary ML classifiers can be trained to discriminate between eligible and ineligible records, calibrated to a threshold score that ensures an acceptably high recall of eligible records. Once calibrated, the ML classifier can then be prospectively tested and applied to subsequent search results to score and then retain or discard records. For (2), a rank-ordered list of records can be continuously reprioritised for manual screening by the same underlying ML classifier, incorporating ‘feedback’ from the growing corpus of manual screening decisions already made about eligible and ineligible records (known as ‘active learning’). Published evaluations of automation tools targeting study selection tasks have typically highlighted a trade-off between marginally higher recall and lower screening workload ^8^.

### The urgent ‘case’ of COVID-19 research

The explosion, during early 2020, in the volume and rate of publication of new primary and secondary research on COVID-19 prompted various efforts to filter and organise this evidence into living maps, specialised registers, or tertiary databases. One example is a ‘living map’, commissioned by the UK Department of Health and Social Care, which we have been maintaining and re-publishing on a weekly basis since February 2020. Up to the end of April 2021, the living map included 52,355 bibliographic records of research articles reporting empirical primary research, modelling studies or systematic reviews on COVID-19, organised into 11 topic codes (based on the main focus of the study). Pre-prints, records of articles not reporting primary data, not relevant to/ not focused on humans, and/or not on the topic of COVID-19, are excluded. From its inception until late October 2020, the map was kept up-to-date using an entirely manual process (detailed further in ‘Methods’). However, it became clear, as publication volume increased, that we would eventually be unable to maintain the map using conventional methods, within available resources. For example, during the first few weeks of producing the map, the information specialist spent 3–4 hours of time-on-task (and up to one day of elapsed time) each week on searching and deduplication tasks. During subsequent months, this increased to 1–2 days of time-on-task (and up to three days of elapsed time), thereby reducing the time available to screen and code records prior to the scheduled publication of each updated version of our living map, at the end of each week. The additional time required was almost all due to the deduplication task taking longer and longer to complete in Endnote, as the number of records increased. Given these threats to sustainability, we started to consider how automation technologies could be used to make search and screening-coding procedures more efficient.

Although there is now a sizeable evidence base for the efficacy of using automation tools in health evidence synthesis, overall uptake remains limited and fragmented. One dimension of the evidence base that is currently lacking is a clearer understanding of any trade-offs between the relative advantages of using automation – potential cost and resource savings – and any impacts on evidence quality. Also, more generally, methods and tools designed to increase the efficiency of evidence synthesis production processes should more routinely be evaluated in terms of their relative efficiency, compared with current standard methods, i.e. in terms of both their costs and effects. In this context, we developed an economic evaluation to assess the costs and effectiveness of automated study identification in our living map of COVID-19 research.

## Methods

### Objective

Our objectives for this work were two-fold. First, the overarching objective was to inform an operational decision about whether we should switch from using conventional Boolean searches of the MEDLINE and Embase databases to using (semi-) automated searches of the MAG dataset, to identify eligible study reports for the living map. Given that the use of the MAG dataset entailed using novel machine learning tools, we also needed to investigate the effectiveness and efficiency of using semi-automated, versus manual, study identification methods to identify eligible study reports.

**Figure 1.**
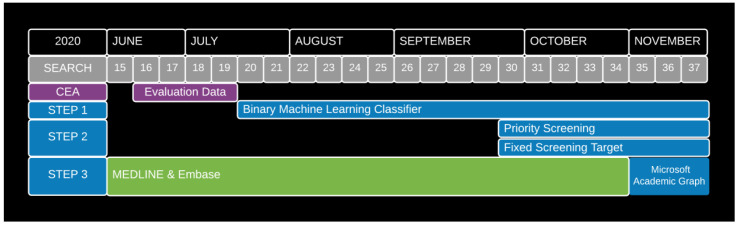
Timeline of automation adoption decisions.

The study was carried out between June 2020 and October 2020. Throughout the period of this study, we moved from a manual workflow to one containing increasing automation. Figure 1 summarises this process, showing that the first automation technology to be deployed was a binary machine learning classifier (from July), followed by using priority screening from the end of September. We made the decision to adopt MAG as our primary source from November. Each of these automation tools is described in detail below following evaluation showing acceptable performance. As Figure 1 shows, the CEA was conducted using data from June / July, before any automation tools were adopted.

This cost-effectiveness analysis (CEA) is reported in line with consolidated reporting standards for economic evaluations ^9^.

We first outline the methods used to find studies in the two sources and then move to describing the comparisons made in the CEA.

### Methods for study identification

***MEDLINE-Embase*.** We searched for potentially eligible records using conventional Boolean searches of MEDLINE (Ovid) and Embase (Ovid) databases each week after their weekly updates. Search strategies are available as extended data ^10^. Retrieved records were downloaded into an EndNote library (version X9) for deduplication between the two sources, followed by deduplication against records retrieved in all previous weeks. Both stages were assisted by EndNote’s semi-automated deduplication tool. The latter tasks were all undertaken by an information specialist. Next, all unique records were uploaded to EPPI-reviewer and manually screened and coded by our team of researchers, with one researcher assessing each record supported by weekly team meetings, where more difficult to appraise records were discussed as a team.

(In practice, weekly searches of MEDLINE and Embase were used to identify records for potential inclusion in the Map from its inception (Search 1 – 4th March 2020 until Search 34 – 26th October 2020 ( Figure 1)). We used screening data, collected from the ‘live’ workflow (using EPPI-Reviewer), covering the period from 22 ^nd^ June to 23 ^rd^ July 2020 (Search 16 to Search 19) to simulate study arms 1 to 5.)

***MAG*.** A new copy of the entire MAG dataset, over 250 million records, arrives in our cloud storage every two weeks. We identify all the new records in each update by comparing it to the previous version. Each update, we build a machine learning model, which distinguishes between relevant and irrelevant records, using the studies that we had included in our map at that point in time. All new records are scored using the model, and top-scoring records are imported into EPPI-Reviewer, where they are semi-automatically deduplicated against records already included in, or excluded from, the living map. (Note that a new deduplication tool was implemented in EPPI-Reviewer during this study; see ‘Study Limitations’ in the Discussion.) In addition to the machine learning model, we also use a Boolean search of MAG embedded in EPPI-Reviewer (called ‘custom Boolean search’). This is a relatively simple search, as it is constrained by the number of characters supported by the search engine (2000) and is detailed in our ‘extended data’ deposited on the Open Science Framework. It relies in particular on two concepts that were automatically added as topic metadata by Microsoft during the pandemic: ‘Coronavirus disease 2019 (COVID-19)’; and ‘2019–20 coronavirus outbreak’. [Update November 2022: these topics have been continued to be added in the OpenAlex dataset.]

(In practice, we switched to using MAG as a single source (replacing MEDLINE-Embase searches) from 9 ^th^ November 2020 (Version 35) onwards. This was the third step of automating our ‘live’ workflow ( Figure 1).)

### Comparisons

We simulated the incremental costs and effects of using eight manual (comparator) or semi-automated (intervention) study identification workflows to maintain our living map for one month between 22 ^nd^ June and 23 ^rd^ July 2020 (map searches 16 to 19 – see Figure 1). This period was selected because it immediately preceded the use of any automation tools in our ‘live’ study identification workflow, thereby providing usable data to measure or simulate the performance of the comparator (manual) workflows.

Components of the eight study-identification workflows (study arms) that we compared are shown in Table 1. Each study arm is described in detail below.

**Table 1.** Characteristics of study arms included in the cost-effectiveness analysis.

	Study arm	Intervention / Comparator	Search	Deduplication between sources	Deduplication against known includes / excludes	Binary machine learning Classifier	Priority screening	Fixed screen Target	Target recall
Manual workflows	1	Comparator A	MEDLINE & Embase	•	•				1.0
	2	Comparator B	MEDLINE & Embase	•	•				0.95
	3	Comparator C	MEDLINE & Embase	•	•			•	0.95
Semi- automated workflows	4	Intervention A	MEDLINE & Embase	•	•	•			0.95
	5	Intervention B	MEDLINE & Embase	•	•	•	•	•	0.95
	6	Intervention C	MAG		•				1.0
	7	Intervention D	MAG		•	•			0.95
	8	Intervention E	MAG		•	•	•	•	0.95

Variations between study arms reflect our primary decision, whether to switch to using the MAG dataset as a single source – and the different scenarios in which this decision could be implemented:

Decision concerning database source:

1. MAG *versus* MEDLINE-Embase.

Decisions concerning automation method:

2. Binary machine learning classifier *versus* none.
3. Priority screening mode *versus* none.
4. Fixed screening target (n = 1,500) *versus* none.
5. Target recall = 1.0 *versus* target recall = 0.95.

‘Comparator A’ (arm 1) corresponds to the baseline workflow that we originally used to create and update our living map; we added two further comparators (arms 2 and 3) into the analysis to enable fair comparisons of workflow performance on each of the five decisions relating to automation processes listed above. The ‘intervention’ workflows (arms 4 to 8) also reflect pragmatic, real-time decisions made by our living map team, regarding which tools we wanted to adopt into our ‘live’ workflow, and when.

### 1. MAG versus MEDLINE and Embase

There are two comparisons to be made in this section. The first concerns whether MAG is sufficiently comprehensive for it to be used as a single source for our searches. The critical question here is the extent to which the records we were identifying through searching MEDLINE and Embase were also on MAG. The secondary question (b) was whether there are records on MAG that we were not finding on MEDLINE and Embase.

To address the first question (a), we assembled a dataset made up of the records identified through our MEDLINE and Embase searches during weeks 16–19 of operation, i.e. before any automation technologies were employed which might have biased the analysis. We then used EPPI-Reviewer (Versions 4.11.4.0 to 4.12.1.0) to semi-automatically match these records against those available in MAG during the same period. This time period was extended by three days to account for the 7- to 10-day lag to the release of the MAG dataset.

To address our secondary question (b: whether MAG contained unique records not found in our MEDLINE and Embase searches), we employed our ‘custom Boolean search’ (see above) of MAG and screened the results using priority screening mode in EPPI-Reviewer (see below). Given the quantity of research being published – and screened – to maintain our formal evidence surveillance pipeline, we only had resource to screen the top 1,500 records (out of the 4,917 retrieved from this search). Thus, while the use of prioritisation to ensure we focused attention on those most likely to be relevant, there may be other relevant records which we did not find. (This means that any benefit found in terms of MAG containing records not found in MEDLINE and Embase may be an underestimate of the true figure.)

The records identified during a) and b) formed the simulation MAG dataset used for study arms 6 to 8.

### 2. Binary Machine Learning Classifier versus None

A binary ML classifier ^11^, designed to distinguish between eligible title-abstract records included in and ineligible records excluded from our COVID-19 living map, was deployed to score new records identified from either the MAG dataset or MEDLINE-Embase databases. Records scoring above a specific threshold score were retained for screening; those scoring below the threshold were discarded. This classifier was calibrated to achieve at least 0.95 recall among MEDLINE-Embase records included in the map, producing a corollary workload reduction of ~30% (compared with screening all MEDLINE-Embase records). This target threshold of 0.95 recall was set in consultation with members of the map team, reflecting our collective willingness to accept a 0.05 reduction in recall among eligible records (i.e., ‘losing’ 5% of eligible studies from the map), compared with screening all search results from MEDLINE and Embase. The classifier used in this simulation study was built in EPPI-Reviewer using MAG records that we had already matched against MEDLINE-Embase records screened until 15 ^th^ June 2020 (Search 15). It was then deployed on MAG and/or MEDLINE-Embase records covering the evaluation period (Search 16 to Search 19), to simulate study arms 4, 5, 7 and 8 ( Table 1).

(In practice, an updated version of this classifier was deployed in our ‘live’ study-identification workflow from 20th July 2020 (Search 20) onwards. This was part of the first step of automating our ‘live’ workflow ( Figure 1).)

### 3. Priority Screening versus None

In EPPI-Reviewer’s priority screening mode, retained records were manually screened for potential inclusion in our living map in prioritised rank order (highest to lowest) based on their scores assigned by the binary ML classifier, described above. The rank order of records awaiting screening was periodically automatically reprioritised based on all preceding eligibility decisions. This approach is known as active learning ^12^. In this study, we simulated the use of priority screening mode on MEDLINE-Embase and MAG records, identified during the Search 16 to Search 19 evaluation period.

(In practice, priority screening mode was used to prioritise the retained MEDLINE-Embase or MAG records for manual screening from 28 ^th^ September 2020 (Search 30) onwards. This was part of the second step of automating our ‘live’ workflow ( Figure 1).)

### 4. Fixed Screening Target versus None

When applying a fixed screening target, manual screening of records in priority screening mode was truncated after a specified target number of records had been screened. In this study and in practice, we specified an overall weekly screening target of 1,500 records. This target reflected the overall quantity of resource (researcher time-on-task) that we decided to expend on manual screening for our living map going forward. Importantly, this was in the context of using machine learning to rank records in order of relevance. In this study, we simulated fixed screening targets in study arms 3, 5 and 8 ( Table 1), using Search 16 to Search 19 data. Without fixed screening targets, manual screening of records continued until the specified target level of recall (see below) was attained among eligible records in the workflow (study arms 1, 2, 4, 6 and 7). For example, when target recall = 1.0, we continued until all records had been screened.

(In practice, we introduced this fixed weekly screening target from 28 ^th^ September 2020 (Search 30) onwards, in conjunction with starting to use priority screening mode. This was, therefore, also part of the second step of automating our ‘live’ workflow ( Figure 1).)

### 5. Target Recall = 1.0 versus Target Recall = 0.95

When target recall = 1.0 (study arms 1 and 6 – Table 1), the objective of the screening workflow is to identify 100% of new eligible records that have been retained from either MEDLINE-Embase (study arm 1) searches or the MAG dataset (study arm 6), to be added to the living map. When target recall = 0.95 (study arms 2 through 5, 7 and 8), this objective is relaxed, and we are willing to ‘lose’ 5% of new eligible records from the living map. In this study, we simulated the specified target recall in each study arm, using Search 16 to Search 19 data.

(In practice, we implicitly adopted target recall of 0.95 when implementing the binary ML classifier from 20th July 2020 (Search 20) onwards. As such, this was also part of the first step of automating our ‘live’ workflow ( Figure 1).)

### Analytic framework

We conducted this cost-effectiveness analysis (CEA) by using a simple decision modelling framework to simulate the incremental costs (resource use) and effects (workflow performance) of using each of the eight variant workflows ( Table 1). We have previously applied the same, transferable model-based economic evaluation framework to assess the cost-effectiveness of using different screening methods in a ‘case study’ systematic review of the effects of undergraduate medical education ^13^.

### Analytic perspective and time horizon

This CEA adopted a single payer perspective because all costs associated with maintaining our living map were incurred by university employers (with income derived from a research grant). The time horizon was four weeks and, therefore, no discount rates were applied to estimated costs or effects.

### Measurement of effectiveness

The outcome measure of benefit was the number of eligible records ‘saved’ from inappropriate exclusion from our living map ^13^. We estimated the recall of each workflow (study arm) against a ‘gold standard’ dataset of eligible (‘include’) records assembled by combining: (i) the total number of ‘includes’ identified from screening 100% of MEDLINE-Embase records (Search 16 to Search 19); and (ii) the total number of includes identified from screening the further set of 1,500 MAG records (out of 4,917 total) identified by our ‘custom Boolean search’ of the MAG dataset (available as extended data ^10^). In addition to the recall of each workflow, we also estimated its precision and corollary impact on screening workload (number of records screened). This analysis was carried out in Microsoft Excel, version 16.51.

### Estimating resources and costs

Resource use was measured as the total time (hours) spent by our information specialists and screen-coding team on completing the manual tasks in each workflow (study arm). For workflows (study arms) that included Boolean searches of MEDLINE and Embase databases, we estimated the time-on-task spent each week by conducting interviews with the information specialists who alternately completed these tasks in our ‘live’ workflow. For workflows that used MAG searches, we recorded the time-on-task spent on deduplication each week after implementing the ‘switch’ to MAG as a single source in our ‘live workflow’. For all workflows, researchers recorded time-on-task spent screening records in our ‘live’ workflow using a simple data collection form (available as extended data ^10^). We then used the collected data to estimate the time-on-task needed to screen 100 records in each specific workflow (base-case analysis) and applied these estimates to the total number of records screened in each simulated workflow, to estimate total time-on-task in hours (total resource use). Although the resources required to initially learn new methods have been cited as a crucial impediment to adoption of automation in this context ^14^, the design of our interventions and comparators introduced no changes that necessitated additional training; we therefore did not measure training time in estimating resources and costs.

An illustrative UK unit cost was obtained from 2020–21 salary scales, published by University College London ^15^. We selected the hourly rate, including London allowance, August 2020 for Spine Point 46, Grade 9 on the UCL non-clinical grade structure. This study was partly funded by a joint UCL-Monash PhD Studentship, and therefore an equivalent illustrative Australian unit cost was selected from Monash University 2020–21 salary scale ^16^, specifically, the mid-point (salary step 4) of academic level B. All cost estimates are, therefore, reported using both 2020 UK GBP (£) and 2020 AUD ($). The total cost of running each workflow was then calculated by multiplying the estimates of total time-on-task (hours) by the UK and Australian unit costs (per hour).

We did not include in our cost estimates:

- The initial time spent developing the Boolean searches for MEDLINE and Embase.
- The time spent by our tech team on incorporating and maintaining the MAG workflow and machine learning models, since this work can now be used in any review in the system.
- Any time spent creating the training dataset for the machine learning model, since this was simply the map itself which required no additional processing.
- Any additional time that the team spent learning any of the workflows evaluated.

### Combining costs and effects

Cost-effectiveness was defined as the incremental cost per eligible study report (record) ‘saved’ from inappropriate exclusion from our living map, compared with current practice, i.e. study arm 1 (comparator A) (i.e. an incremental cost-effectiveness ratio, or ICER ^13^). ICERs could, therefore, in principle, be calculated for any workflow (study arm) that had both higher costs and higher recall, or lower costs and lower recall, compared with current practice (arm 1). If a workflow had both lower costs and higher recall compared with current practice, then this workflow would ‘dominate’ current practice (arm 1) in cost-effectiveness terms, and no ICER would be calculated.

### Analytic assumptions

Our decision to conduct a CEA reflected our interest in achieving a specified unit of output (namely, an eligible study report (record) ‘saved’ from inappropriate exclusion) at the lowest cost, in terms of the resource use associated with this unit of output (effect). Our CEA (base-case analysis) incorporated the following assumptions:

- The inclusion rate (precision) in the study arm 3 (comparator C) workflow, with a fixed screening target, was equal to the overall inclusion rate (precision) observed, in practice, in the study arm 1 (comparator) workflow. The implicit assumption here is that eligible records are distributed at random among a set of bibliographic records screened-coded at quasi-random (i.e. alphabetical order).
- The inclusion rate (precision) in study arms 6 to 8 (interventions C, D and E) workflows, with respect to eligible MAG records also indexed in MEDLINE-Embase, was 0.5. This level of precision is equal to the overall inclusion rate (precision) observed in practice when screening the further set of 1,500 MAG records from the evaluation period identified by our ‘custom Boolean search’ of the MAG dataset. This pragmatic assumption contributed to the simulated overall precision of the arm 6, 7 and 8 workflows in our base-case analysis; and we investigated the impact of varying overall precision, between plausible values, using a sensitivity analysis on arm 8 (described below).
- In study arms 7 and 8 (interventions D and E), the % distribution of hypothetical extra ineligible MAG records between those 'retained' and those ‘discarded’ after deployment of the binary ML classifier was the same as the % distribution of all ineligible MAG records between 'retained' and 'discarded' with respect to Searches 16, 17, 18 and 19 screening data. Again, this pragmatic assumption contributed to the simulated overall precision of the arm 6, 7 and 8 (interventions C, D and E) workflows, in our base-case analysis. The impact of varying precision was investigated using a sensitivity analysis on arm 8 (described below).

### Sensitivity analyses

We conducted two simple, deterministic univariate sensitivity analyses. In the first sensitivity analysis, time-on-task needed to screen 100 records was held constant between the compared workflows. This pre-specified sensitivity analysis was conducted because we judged that variation between study arms, in this measure of resource use, could feasibly be influenced by factors other than the impact of the specified components of each workflow under investigation. In the second sensitivity analysis, we investigated the impact of varying the estimated precision of the study arm 8 workflow (intervention) between plausible lower- and upper-limit values. This post-hoc sensitivity analysis was conducted based on the fluctuating pattern of precision that we observed in practice in our ‘live’ study identification workflow after switching to use of the MAG dataset as a single source (see ‘Results’ and ‘Discussion’). It was conducted on arm 8 because this simulated workflow was closest to the final MAG-enabled workflow that we deployed in practice.

## Results

Our full study dataset, including input data for all parameters, computational formulae and results (summarised below), is available as underlying data ^10^.

### Effectiveness

Table 2 shows the recall (versus gold standard), precision and incremental effectiveness of each simulated workflow (study arms 1-8) (base-case analysis). Compared with workflows incorporating conventional searches of MEDLINE and Embase (arms 1-5), those workflows using MAG as a single source (arms 6-8) had both higher recall and higher precision, saving up to 678 eligible records (arm 6) from inappropriate exclusion from our living map during the four-week study period, as compared with current standard practice (arm 1). These results also show that the use of automation technologies in workflows without MAG increased precision at the cost of reduced recall.

**Table 2.** Effectiveness results.

	Study arm	Comparator/ Intervention	Recall *	Precision **	Incremental effectiveness ***
**Manual**	1	Comparator A	0.83	0.40	-
	2	Comparator B	0.79	0.40	-180
	3	Comparator C	0.55	0.40	-1243
**Semi-automated**	4	Intervention A	0.79	0.55	-167
	5	Intervention B	0.79	0.57	-194
	6	Intervention C	0.99	0.50	678
	7	Intervention D	0.94	0.52	469
	8	Intervention E	0.94	0.86	469

* Recall is the number of eligible records identified divided by the total eligible records from the constructed ‘gold standard’ recall, which included both MAG-identified and MEDLINE-Embase identified eligible records ** Precision is the number of records included divided by the number of records screen-coded ***incremental effectiveness refers to the number of eligible records identified compared to baseline workflow

### Costs

Table 3 shows the resource use, total costs and incremental costs of each simulated workflow (arms 1-8) (base-case analysis). Incorporating use of automation technologies (arms 4-8), fixed screening targets (arms 3, 5 and 8) and relaxed target recall (arms 2-5, 7 and 8) all resulted in lower screening workloads, and therefore, lower total costs, compared with workflows not using these tools and targets. Lower total costs associated with workflows using MAG as a single source (arms 6-8) were primarily driven by eliminating time spent on searching MEDLINE and Embase and deduplication between these two sources, alongside changes in screening-coding workload. In addition, researcher time on task was decreased in semi-automated workflows compared with baseline, demonstrating efficiency gains when using the automation tools of interest. These were not, however, the primary drivers of cost savings (see Sensitivity analyses).

**Table 3.** Cost results.

	Study arm	Comparator/ Intervention	Resource use (hours)	Total cost	Incremental cost
**Manual**	1	Comparator A	234.08	£7,052.72	-
	2	Comparator B	223.18	£6,724.53	-£328.19
	3	Comparator C	158.45	£4,774.01	-£2,278.71
**Semi-automated**	4	Intervention A	150.03	£4,520.48	-£2,532.25
	5	Intervention B	146.71	£4,420.50	-£2,632.22
	6	Intervention C	184.67	£5,564.24	-£1,488.48
	7	Intervention D	168.92	£5,089.56	-£1,963.16
	8	Intervention E	128.57	£3,873.71	-£3,179.01

### Cost-effectiveness

**Figure 2.**
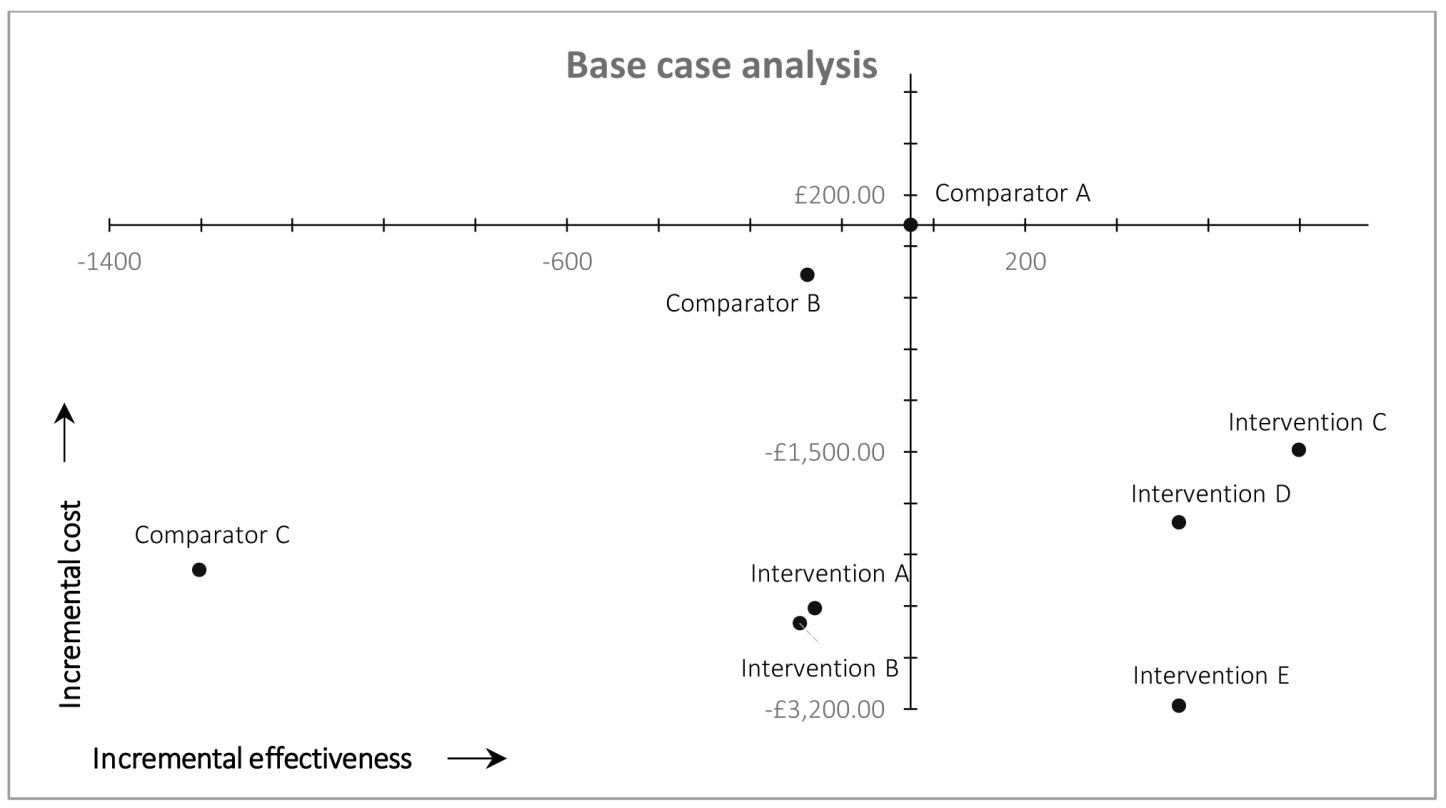
Results of cost-effectiveness analysis (base case).

Cost-effectiveness results (base-case analysis) are plotted on the cost-effectiveness plane shown in Figure 2. Comparator A (arm 1), our original (comparator) workflow, comprising conventional searches of MEDLINE and Embase with no automation or fixed screening targets is plotted at the origin of the cost-effectiveness plane ( Figure 2). The incremental costs and effectiveness of the other workflows are plotted in comparison to Comparator A (arm 1).

All workflows using MAG as a single source (Interventions C, D, and E) are plotted in the south-east quadrant of Figure 2 because they resulted in both higher recall and lower total costs compared with the arm 1 (Comparator A). MAG-enabled workflows, therefore, dominate the original MEDLINE-Embase workflow in cost-effectiveness terms. All other workflows (Comparators B and C, Interventions A and B) are plotted in the south-west quadrant of Figure 2 because they all resulted in lower recall, but also lower costs (due to reduced screening workload and/or higher precision), compared with arm 1. Workflows using automation without MAG (Interventions A and B) resulted in ~4% reduction in overall recall, compared with arm 1, with lower overall costs. Comparators B and C (arms 2 and 3) resulted ~4% and ~28%, respectively, reduction in recall compared with arm 1, again with lower overall costs.

### Sensitivity analyses

**Figure 3.**
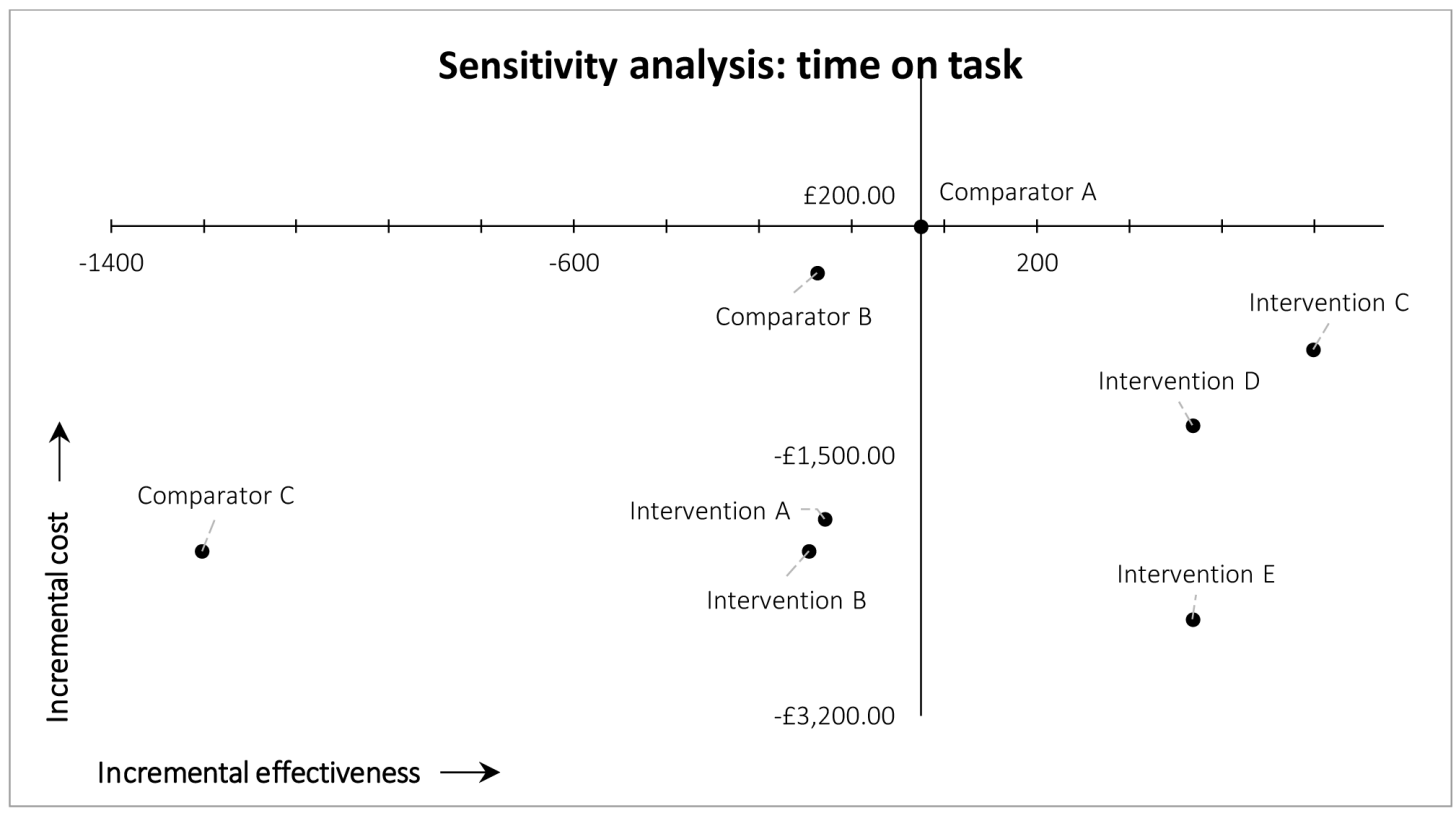
Results of cost-effectiveness sensitivity analysis for time on task.

***Time on task*.** In the deterministic univariate sensitivity analysis in which time on task was held constant between study arms, we used the overall mean time on task among all arms. In this scenario, the overall findings were unchanged: MAG-enabled workflows (Interventions C, D, and E) still dominated current practice (Comparator A/arm 1) (south-east quadrant). The other workflows still resulted in lower recall and lower costs (south-west quadrant) compared with current practice (Comparator A/arm 1). The results of this first sensitivity analysis are illustrated in Figure 3.

**Figure 4.**
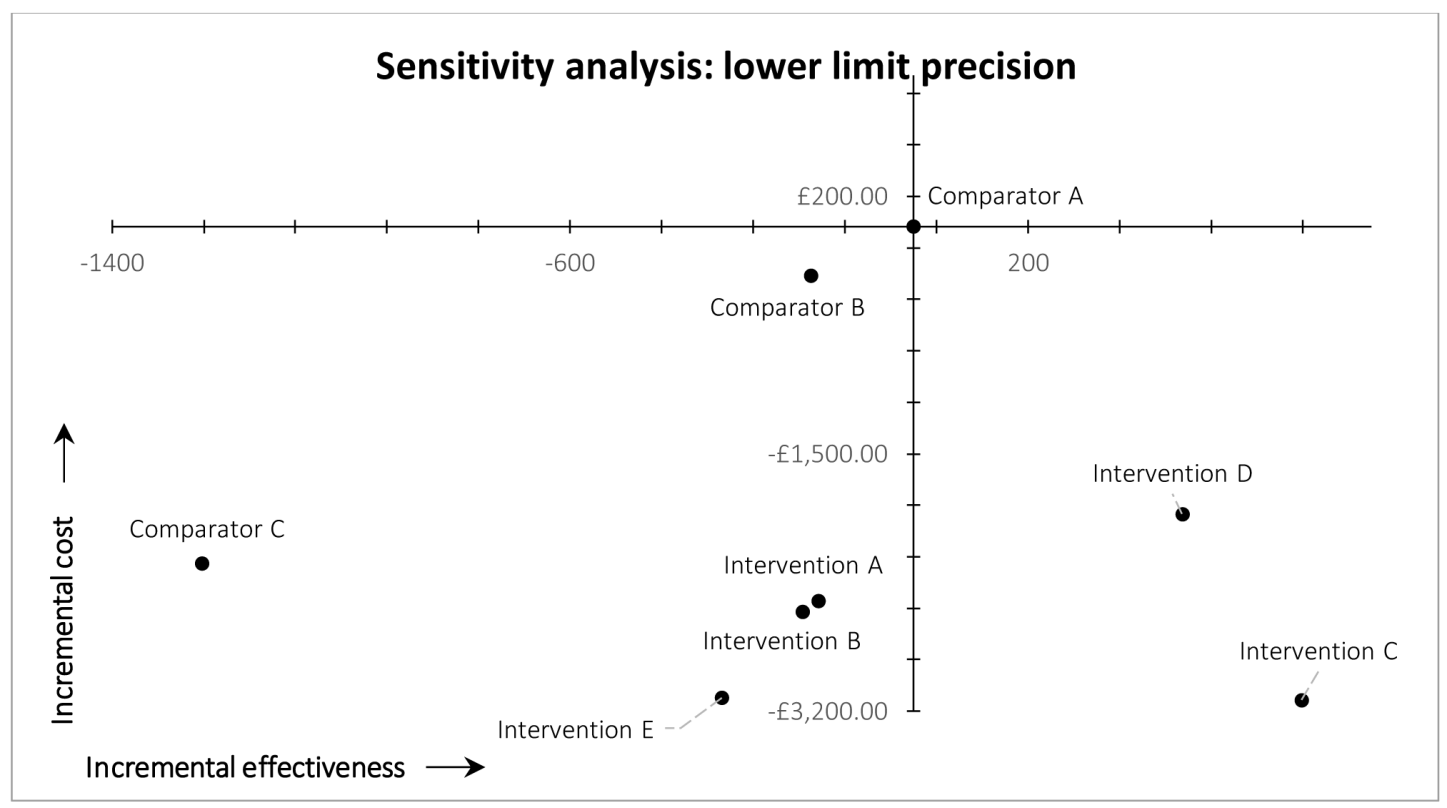
Results of cost-effectiveness sensitivity analysis for precision, lower limit.

**Figure 5.**
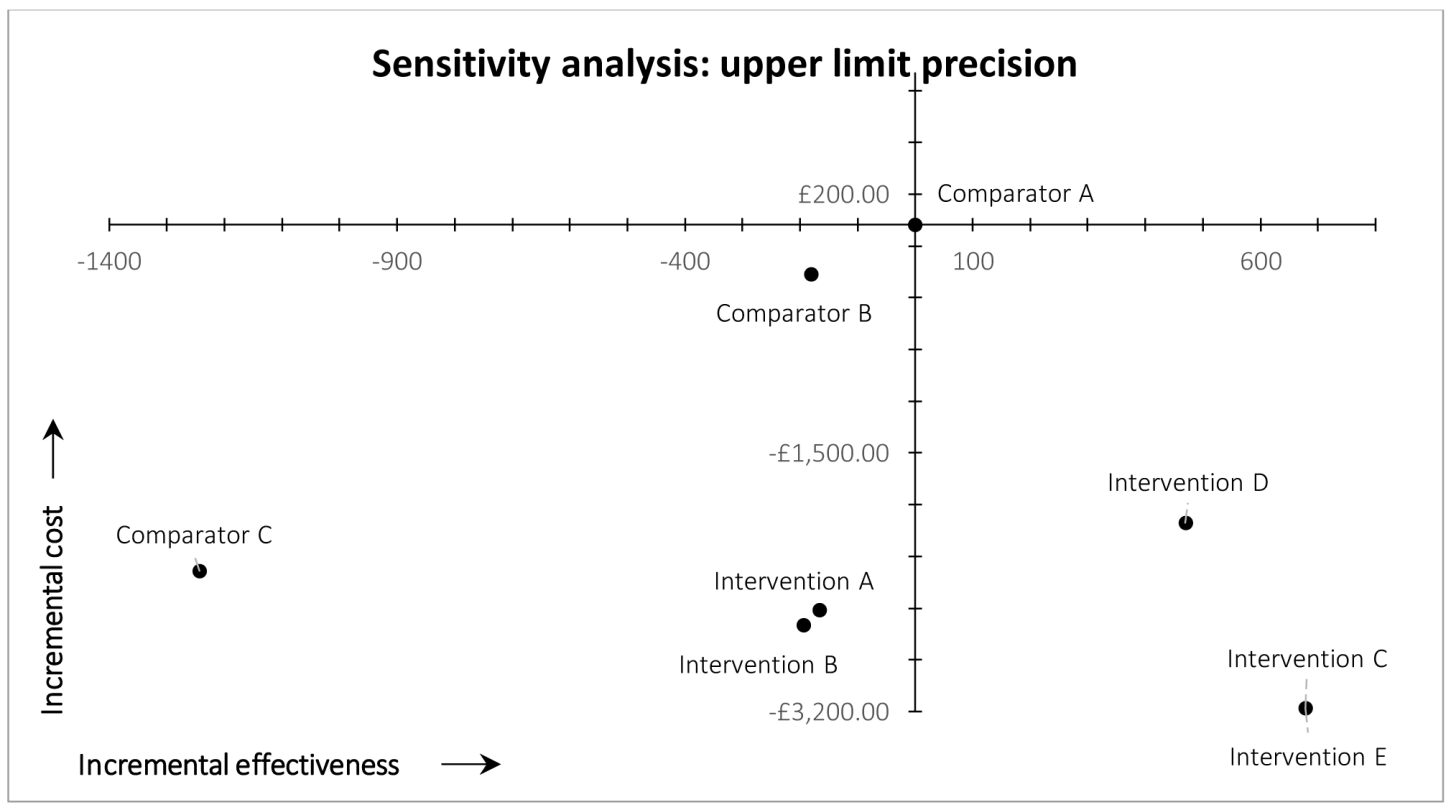
Results of cost-effectiveness analysis for precision, upper limit.

***Precision*.** In the post-hoc deterministic univariate sensitivity analysis on precision ( Figure 4 and Figure 5), we used 0.55 and 0.72 as the plausible lower and upper limit values of precision in the arm 8 (intervention E) MAG-enabled workflow. These values are based on the lower and upper limits of the 95% confidence interval of precision observed in practice in our ‘live’ map workflow, which were 0.55 and 0.74 (see ‘Discussion’). The upper limit value used in the sensitivity analysis is slightly lower than the upper limit of the 95% confidence interval observed in practice, because 0.72 was the maximum precision that could be achieved in the simulated arm 8 workflow (i.e., 4,313 was the total number of eligible records identified by the custom Boolean search of MAG). It was not, however, the maximum precision observed in practice (see ‘Discussion’). Lowering precision to 0.55 inevitably reduced the simulated recall of arm 8 (intervention E); in this scenario the workflow identified 335 fewer records, compared with arm 1 (current practice). This decrease in recall shifted the result for study arm 8 from the south-east quadrant of the cost-effectiveness plane (dominant) into the south-west quadrant (lower recall, lower costs) ( Figure 4). We therefore estimated the threshold level of precision at which arm 8 moved from dominant to non-dominant, versus arm 1 (current practice), which was 0.61.

## Discussion

Given the downstream impacts of evidence outputs – e.g., updated best practice recommendations which impact outcomes for patients, populations, caregivers, and many others – producers of evidence synthesis understandably have rigorous standards for the evidence they produce and disseminate. Therefore, before suggesting changes to standard practice, there is a need to demonstrate that automation of study identification workflows have a relative advantage, compared with current practice for updating living maps, systematic reviews, specialised registers and tertiary databases of research needs. There is also a need to demonstrate alignment with the values and practices of producers of evidence synthesis ^2^. This includes the high value placed on performing a systematic search that attempts to identify all – or more realistically the vast majority – of study reports that would meet the eligibility criteria.

Our simulation was designed to investigate the effectiveness and efficiency of using semi-automated, versus manual, methods for identifying eligible study reports for our living map of COVID-19 research, using a cost-effectiveness analysis framework. The study was primarily undertaken to inform a decision about whether or not to switch to using automated searches of the MAG dataset, replacing the conventional weekly searches of MEDLINE and Embase databases, in our ‘live’ study identification workflow. Our principal findings were that:

- Automated update searches of the MAG dataset had higher recall, compared with conventional MEDLINE-Embase searches;
- Workflows using MAG with automation tools resulted in both higher recall and lower costs, compared with use of MEDLINE-Embase without automation tools; and
- Automation tools alone (without MAG; with or without relaxed screening targets) resulted in both lower recall and lower costs, highlighting the trade-off that is typically seen when deploying such tools to support study selection (screening) in evidence synthesis workflows.

Based on these findings, we decided to adopt a MAG-enabled workflow to maintain our living map of COVID-19 research from November 2020 onwards. The workflow initially incorporated: (i) automated searches of each update of the MAG dataset using our AutoUpdate model and custom Boolean search (see ‘Methods’); (ii) automatic pre-filtering out of MAG records that are pre-prints or from other ‘always excluded’ sources; (iii) semi-automated deduplication of new, top scoring records against existing records already retrieved by previous searches; (iv) application of the binary ML classifier (calibrated to target recall = 0.95); (v) priority screening mode; and (vi) a weekly screening target of 1,500 records.

**Figure 6.**
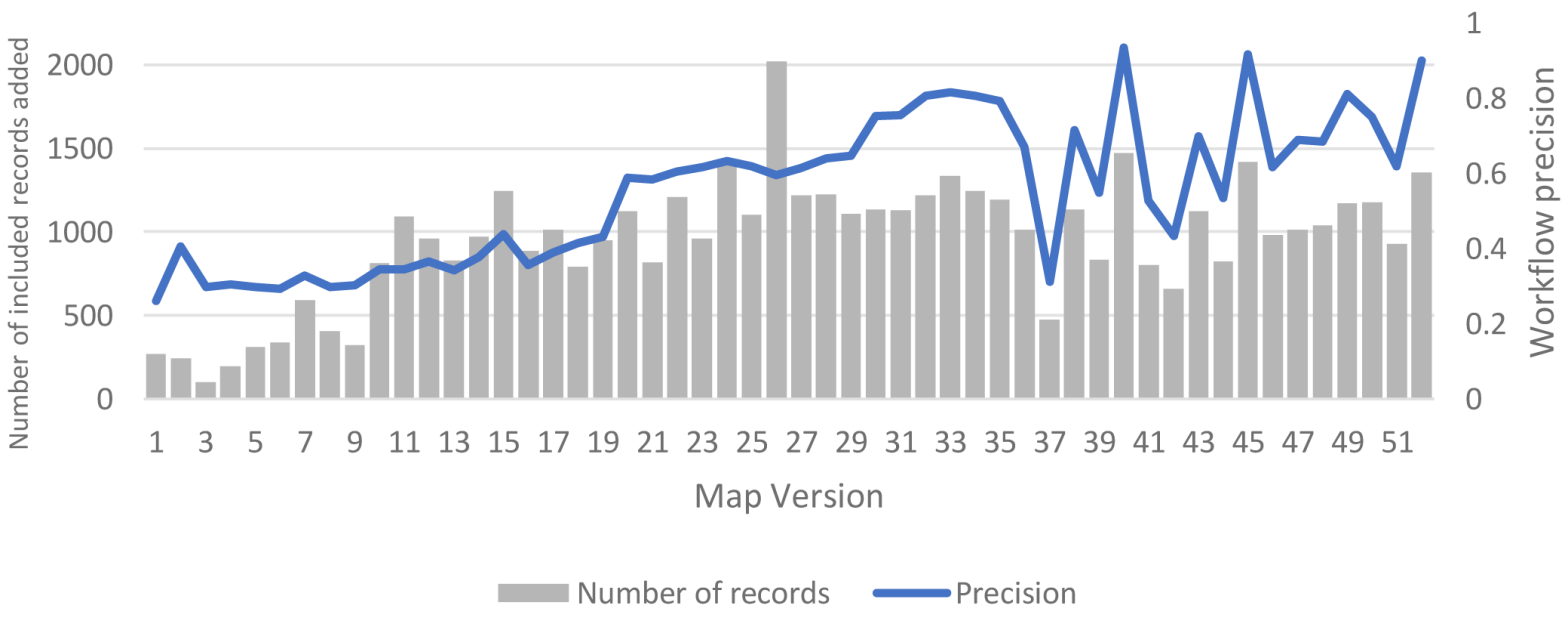
Weekly precision and number of newly included records.

Since adopting the MAG-enabled workflow, we have continued to monitor workflow precision and the numbers of included records (with the fixed 1,500 record weekly target) on a weekly basis. These data are shown in Figure 6, which includes weekly figures for each version of the living map since its inception. As soon as we adopted the MAG-enabled workflow in practice (Version 36), the updating cadence of the MAG dataset lengthened, with a new release approximately every 14 days (instead of the previous every 7 to 10 days). The fluctuating pattern of precision (and numbers of included records) over time that we observed in practice, shown in Figure 6, primarily reflects this lengthened updating cadence: workflow precision is typically high (sometimes extremely high) immediately after new records from each MAG update have been added into the workflow; and then precision falls (often sharply) in the next week(s), before increasing again (often sharply) once new records from the next MAG update have been added.

Up to Version 47 – when we extracted values of the 95% confidence intervals of precision for use in the sensitivity analyses – cumulative precision of the deployed MAG-enabled workflow (with ML tools and a fixed screening target) was 0.65 (95% CI: 0.55 to 0.74); rising to 0.68 (95% CI: 0.60 to 0.75) by Version 52 (12 months after the inception of our living map). Notably, the latter figures are very close to the simulated precision of the arm 8 (intervention E) MAG-enabled workflow in our base-case analysis (0.68), and they are also just above the estimated threshold level of precision (0.61) at which this workflow moved from its dominant to non-dominant position, versus the baseline manual workflow.

Overall, these findings and monitoring data demonstrate the clear potential of our novel workflow, which combines automated searching of the MAG dataset with the use of ML tools, to improve the effectiveness and efficiency of study identification workflows for living maps of research and related evidence synthesis. However, the development and diffusion of this novel approach to searching for and selecting studies is still at a relatively early stage. Further evaluations of the performance of this approach for continuous updating of living maps, specialised registers and tertiary databases of research evidence (as well as for individual systematic reviews), are therefore needed in order to build on and potentially corroborate the promising findings of this study.

### Future research and development priorities

The tools for searching MAG and using the AutoUpdate models are available in EPPI-Reviewer and can be accessed by other teams wanting to explore the potential value of automated searching. We would particularly welcome collaboration with organisations interested in developing and testing MAG-enabled workflows for efficient study identification at scale. For example, we are currently working with the Cochrane Central Executive Team and Cochrane Review Groups to investigate the potential of automated searching of the MAG dataset as a source for: (i) maintaining the Cochrane COVID-19 Study Register; (ii) maintaining Cochrane Specialised Registers of Controlled Trials; and (iii) updating Cochrane (living) systematic reviews. As well as improving the efficiency of Cochrane study identification systems and workflows, our shared aim, with Cochrane, is to reduce the current duplication of effort between our living map of COVID-19 research and the Cochrane COVID-19 Study Register (which, as highlighted in the ‘Introduction’ are two of many overlapping living maps, registers or databases of COVID-19 research evidence currently being maintained globally).

We are also currently undertaking further steps to try to improve the overall precision of our live MAG-enabled workflow, including ‘smoothing out’ the fluctuating precision that we have so far observed in practice ( Figure 6).

One aspect we are aiming to address is the issue of fully non-English titles and abstracts. While screening the set of 1,500 (out of 4,917 total) ‘Search 16 to Search 19’ MAG records identified by our ‘custom Boolean search’ of the MAG dataset for evaluation purposes, we noticed that a non-trivial proportion have fully non-English titles and abstracts; many of these met our map inclusion criteria. We therefore conducted an analysis, based on further manual coding, which revealed that the actual prevalence of fully non-English titles and abstracts among this set of records was 25% (380 of 1,500). This may expose a geographical or language bias in standard databases and suggest that use of a more geographically agnostic dataset could help to overcome this limitation. However, in practice we have observed a much lower prevalence of fully non-English title-abstract records among those MAG records screened-coded in our ‘live’ map workflow since we switched to using MAG as a single source, even after incorporating our ‘custom Boolean search’ strategy (extended data ^10^). Further investigations have revealed this is due to non-English language records being both discarded after scoring by the binary ML classifier (i.e. they fall below the calibrated threshold score) and also de-prioritised in the list of records to be manually screened by active learning, both of which currently use an algorithm that is exclusively based on the text features (and not graph features) of candidate records. We plan to address this limitation in our ‘live’ map workflow by automatically identifying and translating all non-English language records into English language before submitting them for scoring by the binary ML classifier.

Other current priorities for research and development of automation technologies to support study identification in the COVID-map include: the use of Bidirectional Encoder Representations from Transformers (BERT) models for automated assignment of topic codes to eligible (and possibly ineligible) MAG records, and the potential reuse of eligibility decisions made about records screened for the Cochrane COVID-19 Study Register to automatically retain or discard the same records from our living map screening-coding workflow. The latter is another component of our joint initiative with Cochrane, aiming to reduce duplication of effort between workflows used to maintain our living map and the Cochrane COVID-19 Study Register.

### Study limitations

The main limitation of this study is that we were unable to precisely simulate all components of the MAG-enabled study identification workflow that we have subsequently implemented in practice. Specifically, it was not feasible to incorporate use of the AutoUpdate model, which is (prospectively) deployed in automated update searches of the MAG dataset in our ‘live’ workflow, into our (retrospective) simulation. However, this limitation is partly offset by the similar overall levels of performance between our ‘live’ MAG-enabled workflow, which was consistent with the performance predicted by our simulated arm 8 workflow (which was closest to the workflow implemented in practice). Also, the automatic pre-filter that we developed and implemented in our ‘live’ workflow (described above) was developed based on the results of this study, so was unavailable for use during this study.

A second limitation is that, like most model-based economic evaluations, we needed to make explicit analytic assumptions in the absence of data inputs for some model parameters (see ‘Methods’, ‘Analytic Assumptions’). Because this limitation reduces certainty in some model parameters, it could also reduce overall certainty of our model outputs: estimates of costs and effects. However, concern due to this limitation is offset by the consideration that all model parameters were either (i) populated by data inputs collected from our ‘live’ map screening-coding workflow, (ii) underpinned by conservative assumptions, or (iii) underpinned by reasonable assumptions that were tested in our sensitivity analyses.

A third limitation is that, due to limited resources, we decided to stop after screening (for evaluation purposes) the top ranked (using active learning) 1,500 records out of almost 5,000 MAG records identified by our ‘custom Boolean search’ of the MAG dataset (see ‘Methods’). Consequently, our analysis is likely to have underestimated both gold standard recall and the recall of the simulated MAG-enabled workflows against this gold standard, and to have overestimated the recall of the baseline workflow versus the gold standard. However, this limitation also means that our simulated effectiveness (and cost-effectiveness) results, which already show that workflows featuring automated MAG searches had higher recall than those featuring conventional MEDLINE-Embase searches, are likely to be conservative.

It should also be noted that, by the time we deployed the live system, a new deduplication algorithm that is considerably more accurate than that available in EndNote had been implemented in EPPI-Reviewer and was available for use in the live MAG workflow. This will have made the implementation of the work more efficient, as had it been available earlier, the deduplication task would have been less onerous. While this may have had a small impact on the CEA, we have not attempted to quantify the time differential between deduplication in the two environments. This issue does, however, highlight the challenge of adopting and evaluating new technology into evidence synthesis workflows. Tools are undergoing continual development, and it is difficult to determine when the ‘right’ time for evaluation might be. If anything, our experience has found that there is often no ‘right’ time, as algorithms are undergoing continual evolution. What is more important, in our opinion, is that evaluations are carried out, as the evidence base to support the adoption of these new technologies is fairly sparce – despite the explosion in the availability and accessibility of new tools.

Finally, it should be noted that the UK and Australian unit costs that we selected as data inputs to the CEA are illustrative. Selection of higher (or lower) unit costs would have resulted in larger (or smaller) differences between study arms in estimated costs. However, this would not have altered the principal findings of our analysis. We have also separately reported unit costs and quantities of resource use (researcher time-on-task), to enable our results to be recalculated using unit costs applicable to different settings.

## Conclusions

This study has demonstrated the promise of using automated searching of the MAG dataset with machine learning tools to improve the efficiency of living evidence synthesis study identification workflows at scale, by increasing their recall and precision, and reducing production costs.

## Data Availability

Open Science Framework: Cost-effectiveness of MAG and automation for maintenance of a living Covid-19 map. https://doi.org/10.17605/OSF.IO/24W53 ^10^. This project contains the following underlying data: Time on task data.csv (De-identified time on task data collected from the screen-coders) Base case analysis data.csv (All values used in cost-effectiveness calculations) Sensitivity analysis – Precision.csv (All values used in calculations assessing the impact of precision on the cost-effectiveness analysis) Sensitivity analysis – Time on task (All values used in the calculations assessing the impact of time on task on the cost-effectiveness analysis) Open Science Framework: Cost-effectiveness of MAG and automation for maintenance of a living Covid-19 map. https://doi.org/10.17605/OSF.IO/24W53 ^10^. This project contains the following extended data: CHEERS Checklist.pdf (Completed checklist of Consolidated health economic evaluation reporting standards (CHEERS)) Search strategies.docx (Links to MEDLINE and Embase search strategies; MAG custom Boolean search strategy) Time log workbook.xlsx (Workbook provided to screen-coders to collect time on task information) Data are available under the terms of the Creative Commons Zero "No rights reserved" data waiver (CC0 1.0 Public domain dedication). ^1^ There are two publicly available versions of EPPI-Reviewer. An older version, that requires the Silverlight browser plugin to run, and a newer one, that runs in any modern web browser. We refer throughout to the newer version, though the same functions are available in both.
