# Supplementary material for: Cost-effectiveness of Microsoft Academic Graph with machine learning for automated study identification in a living map of coronavirus disease 2019 (COVID-19) research
Source: Wellcome Open Res. 2024 Mar 26;6:210. Originally published 2021 Aug 19. [Version 2] doi: 10.12688/wellcomeopenres.17141.2 (PMC11056680; doi:10.12688/wellcomeopenres.17141.2)
Supplement: Peer review history

# Reviewer report: Reviewer response for version 2

**DOI:** 10.21956/wellcomeopenres.20586.r77706

**Published:** 2024-04-28

**Authors:** Irma Klerings

**Affiliations:** 1 Cochrane Austria, Department of Evidence-based Medicine and Evaluation, Danube University Krems, Krems, Austria

The authors have addressed my concerns regarding the abstract and the methods section. It is now much clearer how the MAG results were generated compared to the Medline-Embase searches, and how resource use was determined.

 As an information specialist, I cannot judge the methodology of the cost-effectiveness analysis.

 The authors also addressed the subsequent switch from MAG to OpenAlex in the introduction and state that results of the study are likely to be transferrable to OpenAlex. However, I think this also needs to be reflected in the discussion, either as part of the limitations or the Future research and development priorities. I also believe the latter section may be in need of updating: Almost 2 years have passed since my original review of this paper. I assume that work on some of the planned steps has since progressed.

Is the work clearly and accurately presented and does it cite the current literature?

Yes

If applicable, is the statistical analysis and its interpretation appropriate?

I cannot comment. A qualified statistician is required.

Are all the source data underlying the results available to ensure full reproducibility?

Partly

Is the study design appropriate and is the work technically sound?

Yes

Are the conclusions drawn adequately supported by the results?

Yes

Are sufficient details of methods and analysis provided to allow replication by others?

Partly

Reviewer Expertise:

Systematic literature searching

I confirm that I have read this submission and believe that I have an appropriate level of expertise to confirm that it is of an acceptable scientific standard.

# Reviewer report: Reviewer response for version 2

**DOI:** 10.21956/wellcomeopenres.20586.r77705

**Published:** 2024-04-01

**Authors:** Su Golder

**Affiliations:** 1 Department of Health Sciences, University of York, York, UK

The authors have addressed the comments made

Is the work clearly and accurately presented and does it cite the current literature?

Partly

If applicable, is the statistical analysis and its interpretation appropriate?

I cannot comment. A qualified statistician is required.

Are all the source data underlying the results available to ensure full reproducibility?

Partly

Is the study design appropriate and is the work technically sound?

Yes

Are the conclusions drawn adequately supported by the results?

Yes

Are sufficient details of methods and analysis provided to allow replication by others?

Partly

Reviewer Expertise:

Information specialist, systematic reviewer and user of automated methods for searching social media

I confirm that I have read this submission and believe that I have an appropriate level of expertise to confirm that it is of an acceptable scientific standard.

# Reviewer report: Reviewer response for version 1

**DOI:** 10.21956/wellcomeopenres.18934.r50095

**Published:** 2022-04-29

**Authors:** Su Golder

**Affiliations:** 1 Department of Health Sciences, University of York, York, UK

Thank you for the opportunity to read this fascinating paper about an important and relevant methodology to increase the cost-effectiveness of study identification.

 Whilst I enjoyed reading this paper I felt that there was a distinct lack of clarity in the section on the different search approaches. In particular, how the MAG results were created, how AutoUpdate worked, what was the MAG custom search, and how MAG worked.

 I feel the paper has greater depth on the CEA analysis than the other methods, whilst this is of interest, I suspect that the majority of the intended audience will be information specialists and systematic reviewers and not health economists. Perhaps the authors could bear this in mind when adding some more clarity.

 I would also like to see mention of the fact that MEDLINE and Embase are both by far the quickest and easiest databases to search and how this automation may be applied to other databases. The content of other databases may not be so well covered in MAG or equivalent. The automation may be more beneficial for tricky to search databases and so forth.

 I think the abstract could better reflect the paper. The emphasis is on multiple databases (when the study was limited to MEDLINE and Embase) seems to overstate this paper. I appreciate this could be relevant for future studies however.

 There appears to be a dearth of references in the introduction. For example, do you have a reference for ‘billions of dollars’, the array of human in the loop automation tools, and the opening up of bibliographic records.

 Can you separate the time for searching and deduplication?

 Can you number the 5 headings (1 to 5) e.g., 1) MAG versus.. 2) Binary Machine Learning etc.

 The limitations could include the constant changing landscape with software, particularly open access software, alluded to in the introduction. Is one researcher assessing each record also a limitation?

 Typo – ‘recall of was’.

Is the work clearly and accurately presented and does it cite the current literature?

Partly

If applicable, is the statistical analysis and its interpretation appropriate?

I cannot comment. A qualified statistician is required.

Are all the source data underlying the results available to ensure full reproducibility?

Partly

Is the study design appropriate and is the work technically sound?

Yes

Are the conclusions drawn adequately supported by the results?

Yes

Are sufficient details of methods and analysis provided to allow replication by others?

Partly

Reviewer Expertise:

Information specialist, systematic reviewer and user of automated methods for searching social media

I confirm that I have read this submission and believe that I have an appropriate level of expertise to confirm that it is of an acceptable scientific standard, however I have significant reservations, as outlined above.

# Response

**Published:** 2024-03-12

**Authors:** James Thomas

**Affiliations:** University College London, UK

Thank you for your really helpful review. And again, apologies for the length of time it has taken us to respond. We have substantially revised the paper, and detail below how we have addressed each of your points of feedback.

- Thank you for the opportunity to read this fascinating paper about an important and relevant methodology to increase the cost-effectiveness of study identification.
  Whilst I enjoyed reading this paper I felt that there was a distinct lack of clarity in the section on the different search approaches. In particular, how the MAG results were created, how AutoUpdate worked, what was the MAG custom search, and how MAG worked. I feel the paper has greater depth on the CEA analysis than the other methods, whilst this is of interest, I suspect that the majority of the intended audience will be information specialists and systematic reviewers and not health economists. Perhaps the authors could bear this in mind when adding some more clarity.

 
*As above in response to Reviewer 1, we have given more information about the MAG dataset and how it was accessed and searched. We have also been through the document (particularly the introduction and methods) and extensively revised our descriptions so that they are clearer for our intended readership.*

 I would also like to see mention of the fact that MEDLINE and Embase are both by far the quickest and easiest databases to search and how this automation may be applied to other databases. The content of other databases may not be so well covered in MAG or equivalent. The automation may be more beneficial for tricky to search databases and so forth.

 I think the abstract could better reflect the paper. The emphasis is on multiple databases (when the study was limited to MEDLINE and Embase) seems to overstate this paper. I appreciate this could be relevant for future studies however. *We have rewritten the abstract and – indeed – the opening sections of the paper in a way that we hope more clearly reflects the analysis. The comparison was MAG versus MEDLINE / Embase, but not only in terms of effort of search (which, in comparison to screening effort, was not large), but in the number of relevant articles found. The results show that, not only is it less effort to search a single source in the way that we evaluated, but that a single source can be more comprehensive than conventional sources too*.

  

- There appears to be a dearth of references in the introduction. For example, do you have a reference for ‘billions of dollars’, the array of human in the loop automation tools, and the opening up of bibliographic records.

*We have rewritten the introductory paragraph without the ‘billions of dollars’ statement. We have also added a reference for the ‘human in the loop’ approach and linked to the relatively recent announcement from Elsevier that it is opening its bibliographic citation dataset.*

  

- Can you separate the time for searching and deduplication?

*We have clarified the split in time required for searching and deduplication to read: …during the first few weeks of producing the map, the information specialist spent 3–4 hours of time-on-task (and up to one day of elapsed time) each week on searching and deduplication tasks. During subsequent months, this increased to 1–2 days of time-on-task (and up to three days of elapsed time), thereby reducing the time available to screen and code records prior to the scheduled publication of each updated version of our living map, at the end of each week. The additional time required was almost all due to the deduplication task taking longer and longer to complete in Endnote, as the number of records increased.*

  

- Can you number the 5 headings (1 to 5) e.g., 1) MAG versus.. 2) Binary Machine Learning etc.

*Good idea – the subheadings are now numbered*.

  

- The limitations could include the constant changing landscape with software, particularly open access software, alluded to in the introduction. Is one researcher assessing each record also a limitation?

*We have added to the discussion some relfections on the challenge of the constantly evolving software landscape. We have also clarified that, while most records were assessed by individual researchers, we had weekly team meetings where the researchers brought the more challenging records for team discussion and resolution.*

  

- Typo – ‘recall of was’.

*Thank you: corrected*.

# Reviewer report: Reviewer response for version 1

**DOI:** 10.21956/wellcomeopenres.18934.r49667

**Published:** 2022-04-25

**Authors:** Irma Klerings

**Affiliations:** 1 Cochrane Austria, Department of Evidence-based Medicine and Evaluation, Danube University Krems, Krems, Austria

This is a very interesting study about using Microsoft Academic Graph within EPPI-Reviewer to update/maintain a living map of COVID-19 research.

 As an information specialist, I cannot judge the methodology of the cost-effectiveness analysis. Instead, I focused on the study identification aspects of the paper.

**Abstract:**

- The abstract does not explicitly reflect two aspects that seem to be crucial to this study: 1) The methodology described was used to (continuously) update an existing evidence map, not to create new map. 2) The study identification methods do not rely on the the MAG dataset alone, but on specific tools implemented in EPPI-Reviewer (AutoUpdate, ML classifiers, priority ranking)

**Methods:**

MAG:

- A major element that is unclear to me is how the MAG results were created in this study. The methods state: “Since it lacks a Boolean search engine, each sequential update of the MAG dataset is automatically searched by deploying our AutoUpdate model. […] To simulate the use of the MAG dataset for study arms 6 to 8, we screened (using priority screening mode in EPPI-Reviewer – see below) the records uniquely identified in the MAG dataset during the evaluation period. To construct this set for screening, we used EPPI-Reviewer (Versions 4.11.4.0 to 4.12.1.0) to semi-automatically match records identified from MEDLINE-Embase searches during the evaluation period against those identified in MAG during the same period.”
- On the other hand, the “gold standard” of eligible records was based on includes of the Medline/Embase search and “the total number of includes identified from screening the further set of 1,500 MAG records (out of 4,917 total) identified by our ‘custom search’ of the MAG dataset.” And the study limitations state: “Specifically, it was not feasible to incorporate use of the AutoUpdate model, which is (prospectively) deployed in automated update searches of the MAG dataset in our ‘live’ workflow, into our (retrospective) simulation.” Based on the methods, I assumed that the MAG records screened for study arms 6 to 8 were identified by AutoUpdate? If AutoUpdate was used, it would be relevant to know how the supervised dataset to train the tool was composed.
- Conversely, if the was the MAG “custom search” feature used this also has to be clearly stated in the “MAG versus Medline and Embase Section”. The authors  contrast their MAG-approach to “conventional Boolean searches”, but the MAG “custom search” is also a type of Boolean search.

Time spent:

- It is not quite clear what tasks were included in the resource use: Apart from recurring tasks necessary for each update search, the Boolean search, the MAG search, and the ML classifiers, would also involve a number of one-time or infrequent tasks, e.g., designing and later adapting Boolean search strategies, creating a supervised dataset for the MAG AutoUpdate, creating and calibrating the ML classifier. Were these included in the analysis? Or does the analysis only include deduplication and screening?
- On a related note, the steep learning curve seems to be a barrier to the use of automation tools in sytematic reviews (Scott, A. M., C. Forbes, J. Clark, M. Carter, P. Glasziou and Z. Munn (2021). "Systematic review automation tools improve efficiency but lack of knowledge impedes their adoption: a survey." J Clin Epidemiol 138: 80-94.) Was the time needed to learn using the MAG search and other tools included in the analysis?

**Discussion:**

- The authors note that the findings of this study are only useful if a MAG-like dataset remains available and is continuously updated. It would be interesting to know if there are already plans to replace MAG with OpenAlex in the EPPI-Reviewer, and whether the authors expect that the cost-effectiveness to remain similar.
- I found the observations concerning non-English studies very interesting. Both the suggestion that there is a certain language bias in the databased commonly used, and that steps have to be taken to avoid that ML and active learning tools exclude/de-prioritize eligible non-English studies are very important considerations. Particularly for research topics of global interest, such as Covid-19.

Is the work clearly and accurately presented and does it cite the current literature?

Yes

If applicable, is the statistical analysis and its interpretation appropriate?

I cannot comment. A qualified statistician is required.

Are all the source data underlying the results available to ensure full reproducibility?

Partly

Is the study design appropriate and is the work technically sound?

Yes

Are the conclusions drawn adequately supported by the results?

Yes

Are sufficient details of methods and analysis provided to allow replication by others?

Partly

Reviewer Expertise:

Systematic literature searching

I confirm that I have read this submission and believe that I have an appropriate level of expertise to confirm that it is of an acceptable scientific standard, however I have significant reservations, as outlined above.

# Response

**Published:** 2024-03-12

**Authors:** James Thomas

**Affiliations:** University College London, UK

Thank you for your very helpful review, and many apologies for the length of time it has taken to respond. We have substantially revised the paper to take account of your feedback. **Abstract:**

- The abstract does not explicitly reflect two aspects that seem to be crucial to this study: 1) The methodology described was used to (continuously) update an existing evidence map, not to create new map. 2) The study identification methods do not rely on the the MAG dataset alone, but on specific tools implemented in EPPI-Reviewer (AutoUpdate, ML classifiers, priority ranking)

*We have updated the abstract to state that: a) the work is concerned with updating living systematic reviews and maps; b) we adapted our systematic review software to incorporate MAG and its associated workflows.*

- **Methods:**
MAG:
- A major element that is unclear to me is how the MAG results were created in this study. The methods state: “Since it lacks a Boolean search engine, each sequential update of the MAG dataset is automatically searched by deploying our AutoUpdate model. […] To simulate the use of the MAG dataset for study arms 6 to 8, we screened (using priority screening mode in EPPI-Reviewer – see below) the records uniquely identified in the MAG dataset during the evaluation period. To construct this set for screening, we used EPPI-Reviewer (Versions 4.11.4.0 to 4.12.1.0) to semi-automatically match records identified from MEDLINE-Embase searches during the evaluation period against those identified in MAG during the same period.”

*We agree this was not sufficiently clear in our original draft. We have now extensively revised this section and state that we received a copy of the MAG dataset every two weeks and used machine learning to identify potentially relevant records each update.*


*  *

- On the other hand, the “gold standard” of eligible records was based on includes of the Medline/Embase search and “the total number of includes identified from screening the further set of 1,500 MAG records (out of 4,917 total) identified by our ‘custom search’ of the MAG dataset.” And the study limitations state: “Specifically, it was not feasible to incorporate use of the AutoUpdate model, which is (prospectively) deployed in automated update searches of the MAG dataset in our ‘live’ workflow, into our (retrospective) simulation.” Based on the methods, I assumed that the MAG records screened for study arms 6 to 8 were identified by AutoUpdate? If AutoUpdate was used, it would be relevant to know how the supervised dataset to train the tool was composed.
- Conversely, if the was the MAG “custom search” feature used this also has to be clearly stated in the “MAG versus Medline and Embase Section”. The authors contrast their MAG-approach to “conventional Boolean searches”, but the MAG “custom search” is also a type of Boolean search.

*We have stated now that we built a new machine learning model each update, trained from the records that were already in the map.*

**

Time spent:

- It is not quite clear what tasks were included in the resource use: Apart from recurring tasks necessary for each update search, the Boolean search, the MAG search, and the ML classifiers, would also involve a number of one-time or infrequent tasks, e.g., designing and later adapting Boolean search strategies, creating a supervised dataset for the MAG AutoUpdate, creating and calibrating the ML classifier. Were these included in the analysis? Or does the analysis only include deduplication and screening?

*We have added a clarification to the section on ‘estimating resources and costs’ to state that we did not include time spent on the Boolean searches, technical development, or creating training datasets.*


*  *

- On a related note, the steep learning curve seems to be a barrier to the use of automation tools in sytematic reviews (Scott, A. M., C. Forbes, J. Clark, M. Carter, P. Glasziou and Z. Munn (2021). "Systematic review automation tools improve efficiency but lack of knowledge impedes their adoption: a survey." J Clin Epidemiol 138: 80-94.) Was the time needed to learn using the MAG search and other tools included in the analysis?

*We agree. We are in the fortunate position of having a technical development and support team within the Centre and can interact directly to describe what is needed. This meant that the machine learning tools were essentially ‘hidden’ from the research team, which simply interacted with the bibliographic data using the standard user interface. We have clarified this in the first paragraph of the “Estimating resources and costs” paragraph and added the reference suggested.*

- **Discussion:**
- The authors note that the findings of this study are only useful if a MAG-like dataset remains available and is continuously updated. It would be interesting to know if there are already plans to replace MAG with OpenAlex in the EPPI-Reviewer, and whether the authors expect that the cost-effectiveness to remain similar.

*This is a very good point. We have added an update to say that* *Microsoft Academic closed at the end of 2021 and the OpenAlex team has picked up its role and it is now the single largest open access repository of scientific bibliographic data. Our EPPI-Reviewer software switched over from MAG to use OpenAlex as its key source of data, and the results reported here for MAG are therefore applicable to OpenAlex too. We have also updated the abstract to clarify this point*


* *

- I found the observations concerning non-English studies very interesting. Both the suggestion that there is a certain language bias in the databased commonly used, and that steps have to be taken to avoid that ML and active learning tools exclude/de-prioritize eligible non-English studies are very important considerations. Particularly for research topics of global interest, such as Covid-19.

*No response needed.*
